# Population density influences genetic connectivity in the Canary Islands endemics *Viola cheiranthifolia* and *Viola guaxarensis*

**DOI:** 10.1093/aob/mcaf317

**Published:** 2025-12-09

**Authors:** Sonia Sarmiento Cabello, Melanie A Murphy, Pedro A Sosa, Alejandro G Fernández de Castro, Jose L Martín-Esquivel, Priscila Rodríguez-Rodríguez

**Affiliations:** Instituto Universitario de Estudios Ambientales y Recursos Naturales (IUNAT), Universidad de Las Palmas de Gran Canaria, Campus Universitario de Tafira, Las Palmas de Gran Canaria 35017, Spain; Department of Ecosystem Science and Management, Program in Ecology and Evolution, University of Wyoming, 1000 E University Ave, Laramie, WY 82071, USA; Instituto Universitario de Estudios Ambientales y Recursos Naturales (IUNAT), Universidad de Las Palmas de Gran Canaria, Campus Universitario de Tafira, Las Palmas de Gran Canaria 35017, Spain; Departamento de Biodiversidad y Conservación, Real Jardín Botánico, CSIC, Madrid 28014, Spain; Parque Nacional del Teide, La Orotava, Tenerife, Islas Canarias 38300, Spain; Instituto Universitario de Estudios Ambientales y Recursos Naturales (IUNAT), Universidad de Las Palmas de Gran Canaria, Campus Universitario de Tafira, Las Palmas de Gran Canaria 35017, Spain

**Keywords:** Alpine ecosystem, oceanic islands, Canary Islands, gravity models, linear models, climate change, conservation genetics, microsatellites, polyploid, *Viola cheiranthifolia*, *Viola guaxarensis*

## Abstract

**Background and Aims:**

Island-endemic plants with narrow geographic ranges are highly vulnerable to invasive herbivores and climate change, which can reduce reproductive success and shift their distributions. To understand how these pressures shape population connectivity, we studied two endemic violets (*Viola cheiranthifolia* and *V. guaxarensis*) from El Teide, Canary Islands.

**Methods:**

We examined genetic diversity and related it to climatic and topographic variables using linear models. To assess how landscape features affect gene flow, we applied gravity models at the population level in *V. cheiranthifolia* (163 individuals) and in 48 *V. guaxarensis* individuals (one population).

**Key Results:**

We found no significant correlation between genetic diversity and environmental variables in *V. cheiranthifolia*. While greater herbivore densities did not appear to decrease connectivity, larger violet populations, including those protected from herbivory, showed greater connectivity. Geographic distance limited genetic exchange in *V. cheiranthifolia*, whereas diurnal temperature changes, precipitation and slope were key factors explaining connectivity in *V. guaxarensis*. Future projections indicated minimal changes in gene flow patterns for both species.

**Conclusions:**

Conservation measures that increase population density enhance genetic connectivity in these endangered endemic violets. Furthermore, understanding how specific landscape variables shape connectivity can directly inform restoration efforts in targeted areas. By identifying key connectivity hubs and zones most in need of intervention, our research offers practical strategies for enhancing the resilience of this unique island flora.

## INTRODUCTION

Habitat fragmentation and climate change are among the most significant drivers of global plant biodiversity loss (Reed and Frankham, 2003; Fischer and Lindenmayer, 2007; Corlett, 2016). In oceanic islands, introduced herbivores contribute to habitat fragmentation by displacing endemic plant populations into smaller, more isolated and marginal habitats, reducing suitable areas for expansion and increasing the risk of local extinction (Cubas *et al.*, 2019). Additionally, herbivores also increase plant mortality rates and reduce flowering in some species, directly impacting fitness (Seguí *et al.*, 2017). These effects are particularly severe on oceanic island species, where native plants evolved without large herbivores and typically lack natural defence mechanisms against introduced grazers (Nogales *et al.*, 2006; Cubas *et al.*, 2019). This vulnerability is exacerbated under climate change, as species are expected to shift their distributions in response to changing environmental conditions (Martín-Esquivel *et al.*, 2021; Hanz *et al.*, 2023). Alpine endemic species in insular ecosystems face additional risks due to limited habitat availability, small population sizes and restricted dispersal capacity, all of which increase their extinction risk (Fernández-Palacios *et al.*, 2021). Given the exceptionally high levels of endemism on oceanic islands (Schrader *et al.*, 2024), understanding the interplay between herbivory, climate change and connectivity loss is essential for developing effective long-term conservation strategies.

Connectivity is a key factor in the long-term persistence of plant populations because it maintains genetic exchange through pollen and seed dispersal, reducing the risk of inbreeding and local extinction (Manel *et al.*, 2003; Frankham *et al.*, 2017). However, ecological and spatial factors such as flowering asynchrony, habitat fragmentation and environmental barriers may reduce connectivity and increase genetic isolation (Auffret *et al.*, 2015; González *et al.*, 2020). Invasive herbivores further contribute to connectivity loss by fragmenting habitats and decreasing plant fitness through higher mortality and lower reproduction rates (Seguí *et al.*, 2017; Martín-Esquivel *et al.*, 2020). In this context, understanding how landscape features and biotic pressures influence connectivity is essential for assessing population resilience in the face of climate change. Landscape genetics offers a powerful approach by linking spatial structure with genetic patterns to reveal how environmental factors shape gene flow and structure (Damschen *et al.*, 2008; Cruzan and Hendrickson, 2020). Genetic data offer direct evidence of realized connectivity, refining habitat permeability models and movement pathways (Razgour, 2015). This integrative framework complements traditional species distribution models (SDMs), which often overlook dispersal constraints and habitat connectivity (Corlett and Westcott, 2013; Aguirre-Liguori *et al.*, 2021). By capturing spatial–genetic patterns, this approach enables a better understanding of species’ responses to global change and has emerged as a key tool in conservation planning for plants (Resasco, 2019; Rico *et al.*, 2023; Sarmiento Cabello *et al.*, 2026).

While alpine ecosystems in continental regions have received considerable attention, high-mountain island ecosystems, such as those in the Canary Islands, remain under-represented in climate and connectivity studies (Courchamp *et al.*, 2014). Mount Teide, the highest peak in Spain, is located within this archipelago and hosts a unique assemblage of alpine flora. Notably, warming on this mountain is occurring at a rate of 0.14 ± 0.07 °C per decade, twice as fast as the rest of the island (Martín *et al.*, 2012). Similar patterns of accelerated warming have been reported on other tropical and subtropical islands (Manes *et al.*, 2021). In response to warming, the flora of Mount Teide has already exhibited upward elevational shifts (Renner *et al.*, 2023) and further distributional changes are expected under ongoing climate change (Hanz *et al.*, 2023). In addition to climatic stressors, this high-mountain ecosystem is also impacted by invasive herbivores, particularly rabbits (*Oryctolagus cuniculus*), which were introduced in the 15th century and are known for their negative effects on the native flora (Nogales *et al.*, 2006; Bello-Rodríguez *et al.*, 2021*b*). Specifically, high-altitude endemic species in the Teide area are particularly vulnerable to rabbit herbivory, even to a greater extent than non-endemic taxa (Cubas *et al.*, 2019; Mancebo *et al.*, 2022). Additionally, alpine shrub habitat, predominant in El Teide, registers higher rabbit densities than other ecosystems within Tenerife (Cubas *et al.*, 2021). Since the declaration of Teide as a National Park in 1954, rabbit populations have been monitored, offering an opportunity to assess their influence on genetic connectivity (Cubas *et al.*, 2022). Moreover, rabbit abundance is negatively correlated with slope, suggesting that steeper slopes may act as refuges from grazing pressure (Cubas *et al.*, 2019).

*Viola cheiranthifolia* and *V. guaxarensis* are dwarf chamaephytic violets endemic to Tenerife Island and found exclusively in Teide National Park. Once considered a single species, genetic and morphological evidence supports their recent divergence and recognition as distinct (Rodríguez-Rodríguez *et al.*, 2019; Marrero-Gómez *et al.*, 2020). Their seeds disperse by explosive ejection, and pollination depends on insects, making connectivity likely influenced by temperature-dependent pollinator activity (Rodríguez-Rodríguez *et al.*, 2019). Despite their geographical proximity, these species occupy distinct high-altitude habitats: *V*. *cheiranthifolia* is found on the slopes of El Teide stratovolcano at elevations ranging from 2400 to 3700 m, while *V. guaxarensis* is restricted to Las Cañadas wall, occurring at around 2600 m (Rodríguez-Rodríguez *et al.*, 2019). Habitat suitability models from previous studies show that temperature, snow cover and precipitation best explain their distribution (Rodríguez-Rodríguez *et al.*, 2019). Climate projections yield conflicting results, with some studies indicating the persistence of suitable habitats (Rodríguez-Rodríguez *et al.*, 2019) while others predict significant habitat loss for both species (Bello-Rodríguez *et al.*, 2023). Additionally, invasive herbivores increase mortality, promote self-fertilization, reduce flowering, and restrict populations to less grazed areas, potentially disrupting genetic connectivity by affecting reproduction and spatial distribution in both species (Seguí *et al.*, 2017; Capó *et al.*, 2024). Despite these pressures, no studies have yet investigated the genetic connectivity of these species or the combined effects of herbivores and future climate conditions on genetic connectivity.

Our main objectives are to (1) evaluate the relationship between genetic diversity and environmental variables; (2) evaluate whether herbivory, climate, topography or spatial distribution influence gene flow in *V. cheiranthifolia* and *V. guaxarensis*, and (3) project changes in functional connectivity under future climate change scenarios.

## MATERIALS AND METHODS

### Studied species

*Viola cheiranthifolia* and *V. guaxarensis* belong to *Viola* sect. *Melanium* subsect. *Bracteolatae* (Marcussen *et al.*, 2022). They share several morphological traits, including compact growth forms (3–6 cm in height) and oval-shaped, hairy leaves (Marrero-Gómez *et al.*, 2020). Both species are perennial and produce chasmogamous, zygomorphic flowers that typically bloom from February to early July, with peak flowering in April–May.

Recent molecular studies revealed two distinct genetic clusters in *V. cheiranthifolia*, leading to the classification of *V. guaxarensis*, found at Guajara summit, as a separate species (Rodríguez-Rodríguez *et al.*, 2019; Marrero-Gómez *et al.*, 2020). Compared with *V. guaxarensis*, *V. cheiranthifolia* shows lower heterozygosity and may be more susceptible to inbreeding. This pattern likely reflects a founder effect and may indicate that *V. cheiranthifolia* populations were colonized by individuals remaining on the Las Cañadas wall after the culmination of the Teide stratovolcano (Rodríguez-Rodríguez *et al.*, 2019). *Viola cheiranthifolia* is classified as Vulnerable (VU D2) in the Red List of Spanish Vascular Flora (Moreno-Saiz, 2008), whereas the conservation status of *V. guaxarensis* has remained unassessed since its recent description (Marrero-Gómez *et al.*, 2020).

Pollination in these species is entomophilous, mediated by over 20 insect species, with *Anthophora alluaudi* and the generalist honeybee *Apis mellifera* being the most common observed pollinators (Seguí Colomar, 2017). Pollinator composition varies with elevation, with greater diversity at lower altitudes (Seguí Colomar, 2017). Despite this, the frequency of pollination interactions is stable across elevations (Lara-Romero *et al.*, 2019). Both species are self-compatible and exhibit ∼50 % autogamy (Rodríguez-Rodríguez *et al.*, 2019). Despite their ability to self-pollinate, cross-pollination has been shown to enhance fruit and seed production (Seguí *et al.*, 2017). In *V. cheiranthifolia*, flowering at higher elevations occurs up to 10 d later than at lower elevations, potentially leading to asynchronous flowering and reduced cross-pollination opportunities. Seed viability is lower at lower elevations, possibly due to increased competition from co-flowering species and heterospecific pollen interference (Seguí Colomar, 2017). According to Yockteng *et al.* (2003), *V. cheiranthifolia* has an approximate haploid chromosome number of 32, although exact estimations of the chromosome number and the ploidy level have not been determined yet.

Dispersal mechanisms have been described in *Viola* as following a diplochory pattern (Beattie and Lyons, 1975), involving explosive seed ejection and potential secondary dispersal by ants (myrmecochory). However, no evidence of myrmecochory has been recorded in the species considered. In addition, both species are hermaphrodite, and their population density was systematically surveyed in a 2019 census, providing baseline data on spatial distribution and abundance within Teide National Park (Docoito Díaz, 2019).

Hybridization between *V. cheiranthifolia* and *V. guaxarensis* is theoretically possible, given the general propensity of violets to hybridize (Marcussen *et al.*, 2022) and that their suitable habitat areas overlap, as documented by Bello-Rodríguez *et al.* (2021*a*). However, no hybrid zones have been identified and no hybrid specimens have been documented. *Viola cheiranthifolia* has not been observed in Guajara, nor has *V. guaxarensis* been recorded in El Teide. Therefore, the potential for successful hybridization remains uncertain, as the absence of observed hybrids may reflect geographic separation rather than biological incompatibility. Moreover, we consider the risk of genetic swamping from other congeners, such as *V. tricolor* and *V. arvensis*, to be very low because these species are geographically distant, occupy different habitat types and have distinct ecological niches and habitat requirements, making their coexistence with the alpine violets unlikely.

### Sample collection and genotyping

**Fig. 1.**
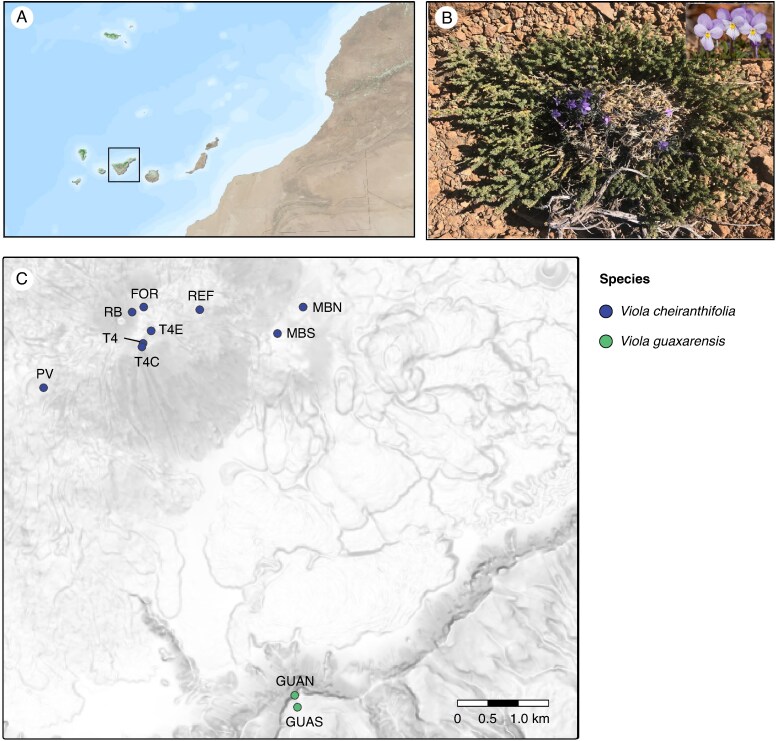
(A) Geographical situation of the Canarian archipelago. Tenerife, the study island, is highlighted. (B) Photograph of *V. guaxarensis* surrounded by *Adenocarpus foliolosus* at Guajara Summit (main) and flowers of *V. guaxarensis*. Photographs by M. Suarez and J. L. Martín Esquivel. (C) Distribution of *Viola cheiranthifolia* (Teide) and *V. guaxarensis* (Guajara). Subpopulations are shown (see Supplementary Data Table S1 for codes). The map background represents slope, with darker areas indicating steeper terrain.

Microsatellite data were obtained from previous studies by Rodríguez-Rodríguez *et al.* (2019) and Rodríguez-Rodríguez *et al.* (2015). Specifically, a total of 163 individuals of *V. cheiranthifolia* from six populations in Teide and 48 individuals of *V. guaxarensis* of a single population in Guajara were sampled. For the purpose of this study, populations were split into subpopulations (Fig. 1, Supplementary Data Table S1). This division was made to provide a more coherent and informative analysis, either because the subpopulations occupied different geographic locations (e.g. MBN and MBS, T4 and T4E) or are subject to different herbivore pressures (e.g. T4C and T4) (Supplementary Data Table S1). Moreover, the *V. guaxarensis* population at Topo de la Grieta consisted of only four individuals with precise GPS coordinates. Given the substantial disparity in sample size compared with the Montaña Guajara population (GUA), this population was excluded from this analysis. Each specimen was georeferenced individually using a Garmin GPS, and fresh leaves were collected and preserved in silica gel. Genomic DNA was extracted from desiccated leaf tissue using the protocol of Dellaporta *et al.* (1983), with a subsequent purification using silica spin columns. Fourteen polymorphic microsatellite markers, previously described for both species (Rodríguez-Rodríguez *et al.*, 2015), were analysed through capillary electrophoresis on an ABI 3130XL, and fragment sizes were determined using GeneMapper v4.0 (Applied Biosystems, Inc.).

### Genetic analysis

In this study we use conservation genetics definitions of gene flow, genetic diversity and genetic distance, focusing on their relevance to population viability and connectivity.

Although subsect. *Bracteolatae* shows high ploidy (Marcussen *et al.*, 2022), a tetraploid pattern has been reported for *V. cheiranthifolia* and *V. guaxarensis* (Rodríguez-Rodríguez *et al.*, 2019). Furthermore, the considered loci showed both auto- and allopolyploid patterns (Sarmiento Cabello *et al.*, submitted).

The analysis of inheritance mode was conducted as part of (Sarmiento Cabello *et al.*, submitted). In brief, we examined *F*_IS_ values, since deviations from expected values suggest tetrasomic inheritance (Meirmans and Van Tienderen, 2012). To further clarify whether deviations from disomic expectations were due to allopolyploidy, we used the testAlGroups function in the polysat package v1.7.7 (Clark and Jasieniuk, 2011) in R to assign alleles to isoloci. This analysis identified three loci of *V. cheiranthifolia* as allopolyploid, which were subsequently excluded from the genetic diversity analysis. Genetic diversity was then calculated using the remaining loci, estimating the average number of alleles (NA) and allelic richness (AR) as the expected number of alleles per locus among *k* gene copies [AR (*k* = 11)] in each population as described by Nei (1972) using SPAGeDi version 1.5 (Hardy and Vekemans, 2002).

Due to the unknown allele dosage typical in polyploid species, genetic analyses such as allele frequency estimation and genetic distance calculations were conducted using methods that account for this uncertainty (e.g. Bruvo distance; Bruvo *et al.*, 2004; Clark and Jasieniuk, 2011). Allele frequencies were first estimated using the simple frequency method and then used to calculate genetic distances based on genotype probabilities. Genetic distance between samples was computed considering all possible unambiguous genotypes and their associated probabilities, assuming 0.5 selfing rate, which has been previously established for this species (Rodríguez-Rodríguez *et al.*, 2019). Using the Bruvo distance metric (Bruvo *et al.*, 2004), the genetic distance between each pair of unambiguous genotypes from two samples was computed and weighted by the product of their respective genotype probabilities. This approach allowed us to account for genotype uncertainty in our distance estimates. These analyses were performed using the polysat package v1.7.7 in R v4.4.0 (Clark and Jasieniuk, 2011; R Development Core Team, 2021).

### Predictor environmental variables

The high resolution of spatial environmental predictors improves the accuracy and performance of niche modelling procedures. For this purpose, the assembly of predictor maps at the fine scale of 20 m per pixel grid based on the LIDAR Digital Elevation Model (DEM) of the Canarian Archipelago (Instituto Geográfico Nacional), restricted to the island of Tenerife, were developed for Rodríguez-Rodríguez *et al.* (2019) and used in this study. In short, the topographic variables slope, northness and topographic position index (TPI) were derived from the DEM using the raster package (Hijmans *et al.*, 2015) implemented in R software. Layers for monthly predictors of minimum, average and maximum temperature and total precipitation were obtained following González Fernández de Castro (2016). The monthly data on precipitation and temperature from 275 climatic stations in the Tenerife agro-climatic network were used as response variables in a stepwise generalized additive model (GAM) using the following predictor variables: (1) for precipitation: altitude, northness and *x* and *y* co-ordinates; and (2) for temperature: altitude, northness, slope and *x* and *y* coordinates, selecting models by the Akaike information criterion (AIC). The residuals of the values of meteorological stations were mapped and interpolated by fixed weighting splines in ArcGIS. The resulting 12 variables for monthly temperature were used to calculate bioclimatic variables following Hijmans *et al.* (2005). Finally, the snow cover layer for Tenerife was also developed for Rodríguez-Rodríguez *et al.* (2019) following Carlson *et al.* (2015) based on snow cover data acquired from Landsat images. For this purpose, higher values were assigned to pixels with greater snow persistence throughout the year, highlighting areas where snow cover is more prevalent and persistent compared with other regions.

At 20-m spatial resolution, even the closest populations of *V. cheiranthifolia* are represented by a substantial number of raster cells. For example, the 1.5-km distance between the PV and RB populations corresponds to 75 grid cells, each with associated environmental information. For *V. guaxarensis*, 36 of the 48 individuals are represented by independent environmental data, ensuring robust coverage of habitat variation (see section Functional connectivity analysis).

### Statistical analysis

A linear regression analysis was performed with the stats package v4.4 (R Development Core Team, 2021) in R to assess the relationship between the predictor variables (Supplementary Data Table S2) and genetic diversity (AR) of *V. cheiranthifolia* populations. Specifically, predictor variables for *V. cheiranthifolia* populations were extracted at the population centroid using the terra package v1.7.83 (Hijmans, 2024). Collinearity between considered variables was assessed using the spatialEco package v2.0.3 (Evans and Murphy, 2021). Variables with high correlation (|*r*| > 0.7) were considered to explain the same patterns to ensure the correct interpretability of the regression model. Because *V. guaxarensis* consists of a single population, this analysis could not be conducted in this species.

### Functional connectivity analysis

#### Gravity models

Gravity models were employed to assess functional connectivity (Murphy *et al.*, 2010). These models are particularly useful to identify landscape features influencing gene flow, even when genetic structure is weak (Dyer, 2015; Rodríguez-Rodríguez *et al.*, 2019). *Viola cheiranthifolia* was represented by nine populations, providing sufficient data to conduct analyses at the population level. In contrast, *Viola guaxarensis* was only present in a single population, and analyses were therefore performed at the individual level. To avoid pseudoreplication, information from individuals located within the same raster cell was aggregated. The coordinates of these individuals were represented by the centroid of their locations, and pairwise genetic distances were summarized by calculating the mean genetic distance between each group and all others.

Gravity models (Fotheringham and O’Kelly, 1989), are based on a network and are uniquely suited to our questions as they incorporate two different types of landscape data potentially influencing connectivity: at-site (network nodes) and among sites (network edges) (Murphy *et al.*, 2010). Gravity models are composed of three parameters: *w* (distance between sites), *v* (at-site variables, production/attraction of flow) and *c* (between-site variables, resistance to flow) (Anderson, 1979). Unlike landscape resistance approaches, environmental variables in gravity models do not need to be converted into landscape resistance.

#### Gravity model parameterization

We calculated *w* as geographic distance between sites and estimated landscape processes influencing production of potential migrants at the site (*v*), following habitat suitability (Rodríguez-Rodríguez *et al.*, 2019). Specifically, snow cover, annual mean temperature (BIO-01) and slope values were extracted using the terra package in R from Instituto Geográfico Nacional (IGN).

For resistance between sites (*c*), variables were selected to ensure that they explained climate (precipitation, temperature) and herbivore access (topography). Herbivore exclusion experiments were not undertaken because only a single population (T4C) is fenced, which prevents a statistically robust comparison between fenced and unfenced conditions. Consequently, topography (slope) was used as a proxy for herbivore pressure, given that empirical work on endemic plants in the study area has demonstrated a negative relationship between rabbit damage and slope (Cubas *et al.*, 2018). Four environmental variables were considered between individuals/populations: maximum mean diurnal temperature range (BIO-02), mean annual precipitation (BIO-12), mean radiation (obtained from Agencia Estatal de Meteorología), mean slope and geographic distance (*k*-nearest neighbour). Gene flow values (1 − Bruvo genetic distance, *T_ij_*), geographic distance and environmental predictors (*w, v* and *c*) were natural log-transformed (ln) for the analysis as follows:


$$T_{ij}=kv_{i}^{\mu }w_{ij}^{\alpha }c_{ij}^{-\beta }$$

$$\ln(T_{ij})=lnk_{i}\;+(\ln\;(\mu v_{i})+\ln\;(\alpha w_{ij})-\;\ln\;(\beta c_{ij}))$$

where *k* is a constant, and parameter estimates are *μ*, *α*, and *β* respectively (Fotheringham and O’Kelly, 1989). To evaluate the appropriate buffer size needed to adequately represent the landscape between sites, we tested multiple buffer zones for each pairwise comparison. This approach allowed us to capture variation across multiple spatial scales and more accurately reflect the environmental conditions influencing connectivity. Specifically, 20-, 60-, 100- and 260-m buffers were applied for *V. cheiranthifolia*, and 20- and 60-m buffers were used for *V. guaxarensis*.

Collinearity was assessed using the spatialEco package in two ways: (1) among parameters within the same buffer to identify non-collinear combinations, and (2) across different buffer sizes for the same variable to determine whether a single buffer provides sufficient representation, based on high correlation with other buffer sizes. For (1), variables with a correlation coefficient |*r*| ≥ 0.7 within the same buffer were considered collinear and one of them was excluded from further analysis. This rigorous screening ensured that only independent variables were included in the construction of full models. For (2), correlation across buffer sizes for the same parameter was used to select a single representative buffer width, assuming high correlation (|*r*| ≥ 0.7) indicates redundancy.

#### Gravity model estimation

Parameters for our set of candidate models for single-constrained gravity models were estimated using the GeNetIt package v0.1.6 (Murphy *et al.*, 2010). Firstly, we ran a null model where connectivity was explained solely by geographic distance between sites (*w*). We tested eight functional connectivity hypotheses, each representing different ecological processes and associated environmental variables (Table 1). The habitat suitability hypothesis considered site productivity, using BIO-01, snow cover and slope, identified as key predictors of habitat quality (Rodríguez-Rodríguez *et al.*, 2019). The establishment hypothesis used the same variables but measured along edges to evaluate conditions facilitating establishment during dispersal. The herbivory access hypothesis was based on the observation that herbivore damage is negatively correlated with slope, as reported by Cubas *et al.* (2018), thereby suggesting that steeper areas experience lower herbivory pressure. The temperature regime hypothesis included mean temperature (BIO-01) and temperature variation (BIO-02) to examine whether populations occurring under different thermal conditions show distinct ecological responses and connectivity. The water availability hypothesis incorporated snow cover and mean precipitation (BIO-12), reflecting the importance of water for germination and survival. The dispersal vector hypothesis included all edge-level variables presumed to hinder or facilitate pollinator and seed establishment, explicitly excluding site-level variables to test whether connectivity is primarily driven by movement conditions rather than production at the site. Finally, we also tested a global model that integrated all variables. A summary of the models and their associated parameters is provided in Table 1.

**Table 1.** List of models and hypotheses tested with parameters at site (*v*) and between sites (*c*) indicated. Abbreviations: mtemp refers to mean annual temperature (BIO-01); tempr refers to mean diurnal range (BIO-02).

Hypothesis	At-site parameters (*v*)	Between-site parameter (*c*)
Habitat suitability	mtemp + snow cover + slope	–
Establishment	–	Precipitation + slope
Herbivory access	Slope	Slope
Pollinator temperature sensitivity	mtemp	tempr
Water availability	Snow cover	Precipitation
Dispersal vector	–	tempr + precipitation + slope
Global	mtemp + slope	tempr + precipitation + slope

#### Gravity model evaluation

Models were ranked using maximum likelihood (Zhang and Callan, 2001) according to the AIC (Akaike, 1973). For the top weighted models, parameter effects were estimated using restricted maximum likelihood (Corbeil and Searle, 1976).

#### Final connectivity graph

To construct the final connectivity graph, we selected the top model along with those having *Δ*AIC < 4. For each model, gene flow values were weighted by each models' Akaike weights, calculated following Wagenmakers and Farrell (2004). Connectivity representation graphs were created using tmap package v3.3.4 (Tennekes, 2018).

### Connectivity in climate change scenarios

To project future climate conditions, we applied the delta method (Hay *et al.*, 2000) to downscale monthly temperature and precipitation anomalies to a 20-m resolution. This downscaling was performed using the downscaleR package in R (Santander Meteorology Group, 2017). We used the CSIRO general circulation model (GCM) for the emission scenario 8.5 developed by IPCC 6 (IPCC Working Group I, 2021) for the year 2080. To assess connectivity under this future scenario, we used the previously estimated gravity model coefficients to predict future values by applying them to updated climatic inputs (temperature and precipitation anomalies), while holding topographic variables constant. All climatic predictors were transformed (e.g. log-transformed) consistent with the original model structure to ensure compatibility of prediction inputs. To compare present and future conditions, we calculated the difference between future and present scenarios using the terra package in R. This approach enabled us to spatially quantify projected environmental changes across the study area.

## RESULTS

### Objective 1: higher genetic diversity relationship with temperature and water availability

**Fig. 2.**
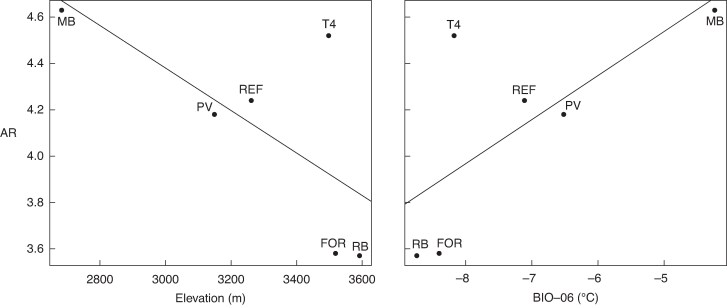
Relationship between allelic richness (AR) and two environmental variables across six populations of *Viola cheiranthifolia*: (Left) Elevation and (right) minimum temperature of the coldest month (BIO-06). Points represent population means of AR, and regression lines indicate the linear relationship between AR and each environmental variable.

We evaluated the relationship between genetic diversity and 23 environmental variables (Supplementary Data Table S2). Radiation and minimum temperature of the coldest month (BIO-06) showed among the strongest linear associations with genetic diversity (Fig. 2, Supplementary Data Table S3). In general, populations at lower elevations, which experience higher evapotranspiration and warmer minimum temperatures, also exhibited higher genetic diversity (Fig. 2). An exception was population T4, which maintained relatively high genetic diversity compared with other high-elevation populations, such as RB and FOR. However, none of the tested variables showed a statistically significant relationship with genetic diversity (*P* > 0.05; Supplementary Data Table S3).

### Objective 2: connectivity and environmental variable effect on gene flow

#### Viola cheiranthifolia

**Fig. 3.**
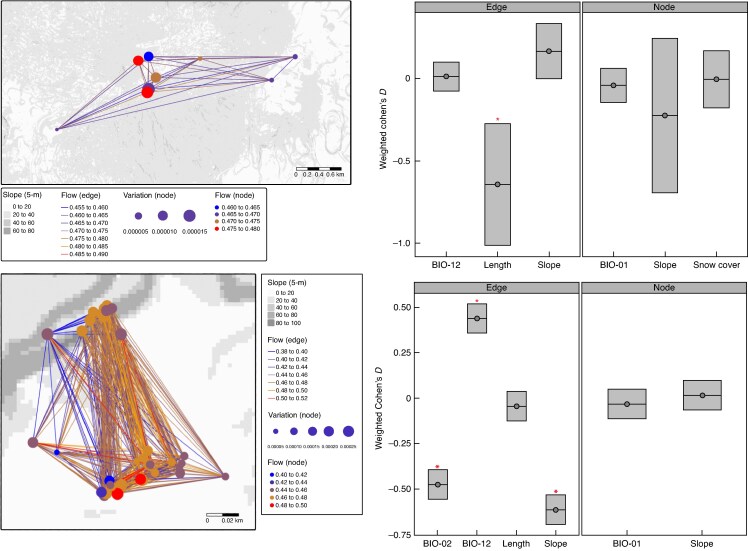
Functional connectivity with a 20-m buffer for *V. cheiranthifolia* (top) and *V. guaxarensis* (bottom). (Left) Network maps where edge colour denotes pairwise gene flow (flow; 0–1), node colour shows mean gene flow per node (0–1), and node size scales with within-node variation (variation). The top map shows nine subpopulations; the bottom map shows 36 groups/individuals. The background raster depicts terrain slope (darker tones = steeper areas), derived at 5-m resolution from a digital elevation model (DEM) provided by the Instituto Geográfico Nacional (IGN), accessed via https://martingonzalez.net/ign-dem-grabber/. (Right) Standardized directional effects of environmental predictors from top-ranked models (Table 1); values >0 indicate increased gene flow and values <0 indicate reduced flow. Asterisks mark effects whose 95 % confidence intervals exclude zero (considered significant).

Gene flow values between populations ranged from 0.455 to 0.490 (Fig. 3). Populations T4C and RB exhibited the highest gene flow values, while populations at lower elevations displayed the lowest gene flow values (Fig. 3, Supplementary Data Table S6). Site parameters were highly correlated across buffer sizes (|*r*| > 0.75), except for between-site slope (*c*) between the 20- and 260-m buffers (|*r*| = 0.649; Supplementary Data Table S4). Given this, we retained the 20-m buffer as the primary spatial scale for analysis. However, we also ran models using the 260-m buffer to evaluate whether results differed substantially. As shown in Supplementary Data Table S4, the outcomes were consistent across both scales. Within the 20-m buffer, none of the parameters were collinear (|*r*| ≤ 0.68; Supplementary Data Table S4) and populations did not overlap within the same raster cells.

Five models scored *Δ*AIC < 4 (Table 2). The null model ranked highest, followed by the herbivore access model. Additional statistical details for all models are provided in Supplementary Data Table S5. Among environmental predictors, only geographic distance between populations showed a negative effect on gene flow (*d* = −0.67, 95 % CI −1, −0.3, Supplementary Data Table S7), indicating that greater distances are associated with reduced gene flow (Supplementary Data Table S6, Fig. 3). All other predictors, including mean annual temperature, had confidence intervals that included zero, suggesting no clear effect on gene flow (Supplementary Data Table S6, Fig. 3). The best-performing models did not include diurnal temperature ranges or radiation, and therefore no data were generated to evaluate their effect on connectivity.

**Table 2.** Model selection results for two species of *Viola* (*V. cheiranthifolia* and *V. guaxarensis*) based on the AIC. For each model, the AIC value (AIC), the delta AIC (*Δ*AIC) and the Akaike weight (wAIC) are reported.

Model	AIC	*Δ*(AIC)	*w*(AIC)
*Viola cheiranthifolia*			
Null	−349.951	0	0.416
Herbivory access	−349.162	0.79	0.281
Establishment	−348.306	1.65	0.183
Habitat suitability	−346.12	3.83	0.061
Water availability	−346.052	3.89	0.059
*Viola guaxarensis*			
Dispersal vector	−2494.79	0.001	0.837
Global	−2491.51	3.23	0.163

#### Viola guaxarensis

Gene flow values between individuals ranged from 0.38 to 0.52 (Fig. 3). Individuals from the southern sector of Guajara had more pairwise connections with higher gene flow values than the northern section. Variables were highly correlated across buffers (|*r*| > 0.9), so the 20-m buffer was chosen for subsequent analysis to reduce redundancy. At-site snow cover and slope were collinear (|*r*| = 0.87; Supplementary Data Table S4). Slope was retained because it is directly related to one of the main hypotheses in this study, testing how topography may mediate herbivore pressure, which is known to influence population persistence in alpine environments. Likewise, mean slope and mean radiation showed strong collinearity (|*r*| = −0.98), and slope was again prioritized, as it offers a more direct link to dispersal and herbivory-related processes. Furthermore, 19 individuals shared the same raster cell. To avoid pseudoreplication, their data were aggregated (see Materials and methods section), reducing the dataset from 48 individuals to 36 unique points, 7 of which represent combined data from multiple individuals.

Two models scored *Δ*AIC < 4 (Table 2). The top-ranking model was the dispersal vector hypothesis model, followed by the global model. The null model had a substantially higher AIC value, indicating lower support for models including environmental predictors. Additional model statistics are presented in Supplementary Data Table S5.

In *V. guaxarensis,* steeper slopes and higher diurnal temperature variation were associated with reduced gene flow. In contrast, precipitation was associated with increased gene flow (Supplementary Data Table S7, Fig. 3). However, the actual differences in environmental values were small, with a maximum change of 11.36 mm in precipitation and 0.03 °C in temperature. Geographic distance did not show a clear effect on gene flow, as its confidence interval included zero (*d* = −0.073, 95 % CI −0.121, 0.038; Supplementary Data Table S7). Other variables showed no clear effect, as their confidence intervals also included zero.

### Objective 3: connectivity under future climate change scenarios

**Fig. 4.**
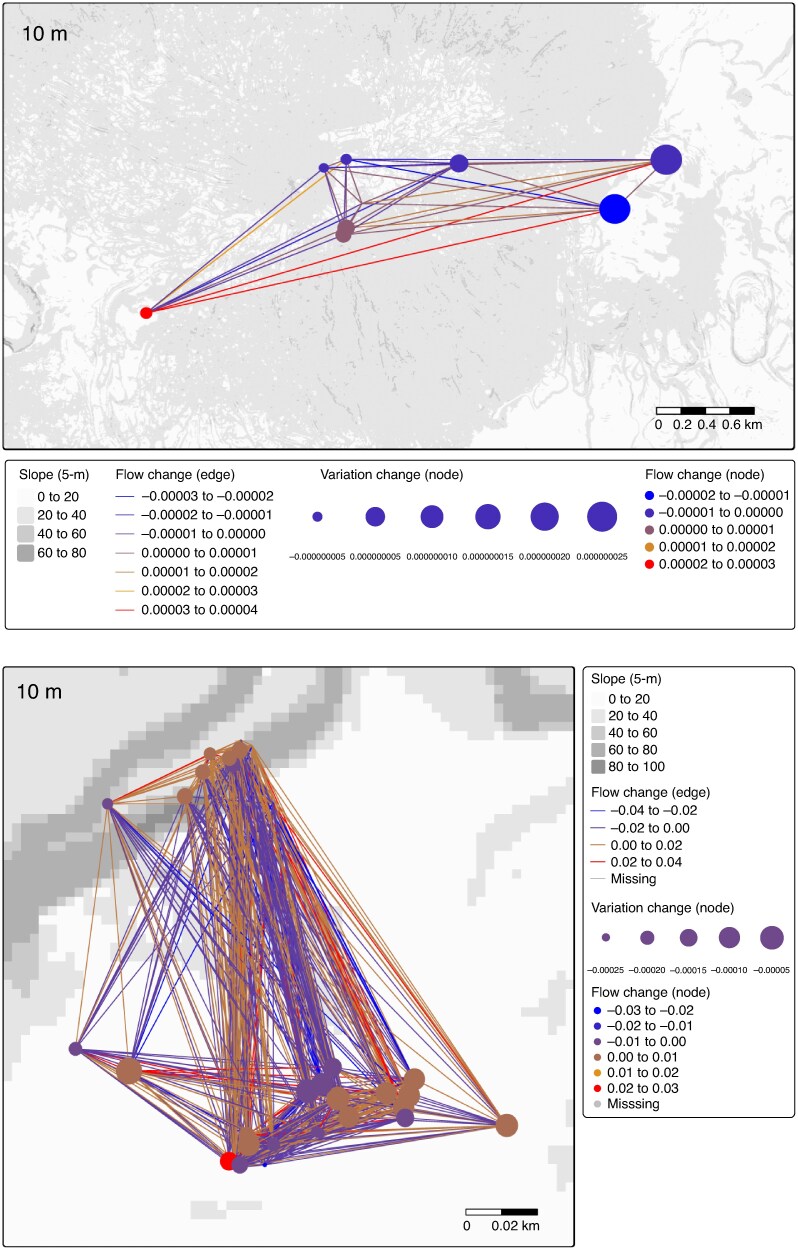
Change in functional connectivity between present conditions and the 2080 RCP 8.5 scenario for *V. cheiranthifolia* (left) and *V. guaxarensis (*right), computed with the 20-m buffer. Edges are weighted by the change in pairwise gene flow (flow change = future − present; range −1 to 1). Node colour shows the change in mean gene flow per node (flow change, 0–1 → −1 to 1), and node size scales with the change in within-node variation (variation change). Positive values indicate an increase in gene flow or variance under the future scenario, while negative values reflect a decrease. The background raster depicts terrain slope (darker tones = steeper areas), derived at 5-m resolution from a digital elevation model (DEM) provided by the Instituto Geográfico Nacional (IGN), accessed via https://martingonzalez.net/ign-dem-grabber/.

Projected climate scenarios indicate minimal changes in gene flow compared with current conditions (Fig. 4, Supplementary Data Fig. S1). Changes in gene flow were much smaller for *V. cheiranthifolia*, with deviations roughly 100 times smaller than those for *V. guaxarensis*, where changes ranged within ±0.03. Overall, the direction of change is uncertain: there were slightly more pairwise connections between individuals predicted to show reduced gene flow (662) than increased flow (598).

## DISCUSSION

Maintaining and enhancing spatial connectivity in endangered species is one of the central targets of nature conservation. We examined genetic diversity and gene flow in two endemic violet species from the high-mountain ranges of El Teide National Park. *Viola cheiranthifolia* populations at lower elevations showed higher genetic diversity. However, no significant associations with environmental variables were found that could explain differences in genetic diversity. Populations of *V. cheiranthifolia* and *V. guaxarensis* show homogeneous gene flow across the landscape, with slightly higher levels in larger populations. Although the two species are closely related, the environmental factors influencing gene flow differ between them. Notably, our analysis predicts only minor shifts in connectivity under climate change conditions.

### Genetic diversity altitudinal pattern may reflect past colonization events

Genetic diversity in *V. cheiranthifolia* did not show significant relationships with any environmental variable. This may be due to the limited number of extant populations at the time of sampling (*n* = 6), resulting in low statistical power. Despite this, a genetic pattern is apparent: genetic diversity tends to increase with lower elevation. Specifically, lower-elevation populations, which also experience higher temperatures, exhibit greater genetic diversity. One possible explanation for this pattern relates to pollination. *Viola cheiranthifolia* relies on insect pollinators, which are ectothermic organisms whose activity depends on temperature (Rodríguez-Rodríguez *et al.*, 2019). At higher elevations, lower temperatures may reduce pollinator activity duration, thereby limiting gene exchange between individuals and leading to decreased genetic diversity. However, and despite evidence of reduced pollinator abundance and diversity at higher elevations in El Teide (Lara-Romero *et al.*, 2019), no decline in pollination interactions has been observed at higher elevations (Seguí Colomar, 2017). In fact, at the Teide summit *V. cheiranthifolia* remains the only species reliant on entomophilous pollinators (Lara-Romero *et al.*, 2019), making it unclear whether pollination constraints significantly contribute to the observed genetic diversity patterns at this time.

Another possible explanation lies in historical climatic changes. During the postglacial period, higher elevations were covered in snow and ice for longer periods than they are today (Glas, 1982; Moreno, 2010). During this time, lower-elevation populations may have served as genetic reservoirs, maintaining greater genetic diversity, as previously well established in other species (Holderegger and Thiel-Egenter, 2009). As climate conditions shifted, recolonization of higher altitudes from these lower-elevation populations may have led to a ‘leading-edge’ expansion, where only a subset of individuals with lower genetic diversity migrated upwards. This recolonization hypothesis is supported by historical records indicating that violets once historically occurred at lower elevations on El Teide than where they are currently found (Renner *et al.*, 2023), and is also consistent with ongoing patterns of plant migration driven by climate change on El Teide (Martín Esquivel and Pérez González, 2019). Moreover, similar postglacial recolonization patterns have also been observed in other high-mountain species (Morente-López *et al.*, 2018; Reisch and Rosbakh, 2021).

Additionally, the findings on genetic diversity do not follow the central–marginal model, which predicts higher genetic diversity in core populations due to larger size, greater gene flow and higher stability, while peripheral populations are expected to be smaller, more isolated and genetically depauperate (Kawecki, 2008). *Viola cheiranthifolia* genetic patterns do not align with the expectations of the central–marginal model as lower-elevation populations have greater genetic diversity than those at higher elevations. This further supports the hypothesis that historical climate shifts and demographic events, rather than current conditions, may have shaped this species’ genetic diversity. However, additional populations will need to be included to strengthen this inference, as the relationship between genetic diversity and temperature was not statistically significant.

### Key areas for connectivity are shaped by population density

Despite the relatively high selfing rate in *V. cheiranthifolia* (∼0.5; Rodríguez-Rodríguez *et al.*, 2019), the observed genetic distance values (>0.5) indicate effective outcrossing that maintains genetic variation (Seguí *et al.*, 2017; Rodríguez-Rodríguez *et al.*, 2019). Experimental evidence supports this pattern, as seed set per capsule is significantly higher under open pollination than under selfing (Seguí Colomar, 2017). Although overall variation in gene flow among *V. cheiranthifolia* populations is limited, two populations show slightly increased connectivity, while *V. guaxarensis* displays higher gene flow within the southern sector of the Guajara population.

The most interconnected *V. cheiranthifolia* populations are found at high elevations, which likely reflects recent colonization of high-altitude sites on El Teide (Renner *et al.*, 2023). Specifically, historical records document the species up to 3400 m in the 19th century, indicating populations at higher elevations (RB, FOR, T4) are recent establishments following upward range expansion. Among these, RB, the highest population sampled, shows high gene flow but low genetic diversity, consistent with a founder effect. Therefore, ongoing and recent gene flow from surrounding populations likely maintains its connectivity. In contrast, FOR, a similarly recently established population at high elevation, exhibits lower connectivity, potentially reflecting its smaller population (Docoito Díaz, 2019).

Differences in density also explain patterns between T4 and T4C, two adjacent populations differing in herbivory exposure. T4C, which is fenced, exhibits higher plant density and larger individuals due to herbivore exclusion (Docoito Díaz, 2019). This is consistent with prior findings that herbivore exclusion increases flower and plant density, as well as outcrossing rates (Seguí *et al.*, 2017). Consequently, herbivore exclusion may indirectly enhance connectivity by promoting higher population density and reproductive success, a process of growing relevance given predictions that rabbit populations will expand upwards in El Teide with climate change (Bello-Rodríguez *et al.*, 2021*b*). Nonetheless, this mechanism should be tested experimentally by using replicated exclusion and control plots to confirm the extent to which herbivore removal directly influences connectivity. Additionally, phenological shifts were also considered as a potential factor affecting connectivity. In *V. cheiranthifolia*, flowering occurs later at higher elevations, with a lag of ∼10 d relative to lower-elevation populations (Seguí Colomar, 2017). However, given that the species’ flowering period can extend from February to June, this variation is unlikely to generate reproductive isolation.

In *V. guaxarensis*, higher connectivity in the southern sector of Guajara cannot be attributed to differences in herbivore density, flowering asynchrony, pollinator density or morphology, as these variables are homogeneous across sectors, likely due to their spatial proximity and homogeneous environmental conditions. The only distinguishing factor is the higher density of individuals in the southern area (J. L. Martín Esquivel, pers. comm.). Therefore, the most plausible explanation for the enhanced gene flow is higher population density, which increases the likelihood that pollen and seed are successfully dispersed. This pattern parallels that of *V. cheiranthifolia*, although stochastic demographic processes, such as founder effects, may also contribute.

Overall, our findings indicate that population density is the primary determinant of genetic connectivity in both *Viola* species. Larger populations exhibit higher gene flow, a pattern consistent with that observed in other taxa (e.g. Aavik *et al.*, 2014). Mechanistically, denser flowering patches attract more pollinators and function as key pollen sources (Richards *et al.*, 1999; Dauber *et al.*, 2010), while producing greater numbers of seeds with higher dispersal probability (Ellstrand and Elam, 1993). Furthermore, seeds from larger populations also tend to show higher germination success and fitness (Faast *et al.*, 2011). Hence, increased population density, whether through natural expansion or management interventions such as herbivore exclusion, appears to enhance both pollination-mediated and seed-mediated gene flow, sustaining the long-term connectivity and viability of high-mountain *Viola* populations.

### Herbivore predation effect is not observed in current connectivity patterns

Although the herbivory exclusion model ranked among the highest-performing models for *V. cheiranthifolia*, slope did not have a statistically significant effect on genetic connectivity. These findings suggest no detectable effect of slope on genetic connectivity, either because the effect is negligible or because our analyses lacked sufficient power to detect it. Furthermore, at Mount Teide, herbivory density is high at Pico Viejo and lower in Montaña Blanca (Cubas *et al.*, 2022). Despite this, gene flow levels are similar in both populations, with *V. cheiranthifolia* connectivity remaining comparable under varying rabbit pressures. At Guajara Summit, the herbivory exclusion model did not rank among the top models in *V. guaxarensis*, indicating that herbivory does not appear to shape current connectivity patterns.

Overall, we found no evidence that predation from invasive herbivores reduces genetic connectivity in either violet species. This contrasts with findings for other single-island endemic species in the Canary Islands, which have shown altered genetic composition as a result of habitat fragmentation driven by herbivore predation (Rodríguez-Rodríguez *et al.*, 2024). One possible explanation is that we may be overlooking the impact of other herbivores on violets. In Teide National Park, mouflons (*Ovis gmelini*), introduced for hunting, also negatively affect endemic vegetation. However, dietary studies have not identified violets in their forage (Nogales *et al.*, 2006), supporting the view that rabbits may exert the strongest grazing pressure on these species. Alternatively, the negative impact of rabbits on violet fitness may not be strong enough to produce detectable differences in gene flow. This may be due to insufficient time for neutral genetic differentiation to reach equilibrium, given the long generation times (∼25 years), overlapping generations and limited seed dispersal of *Viola* species, all of which can delay genetic response to ecological pressures (Epps and Keyghobadi, 2015). Another possible explanation is that herbivore density varies seasonally. In spring, herbivore density is lower compared with summer (Cubas *et al.*, 2022), and this reduced density coincides with the peak flowering period of the violets in April, with some individuals even beginning to flower as early as February. In contrast, high herbivore density in summer overlaps with the final months of the flowering period, a time point when gene flow may have already taken place.

In Guajara, *V. guaxarensis* grows within the Canary Island flatpod (*Adenocarpus foliolosus*), which provides protection from herbivores (Fig. 1B). This shrub contains a natural alkaloid that acts as a herbivore deterrent, shielding the violets from grazing (Irl *et al.*, 2012). Initially thought to represent a purely biotic relationship (Wildpret de la Torre and Martín Osorio, 2005), the association between *V. guaxarensis* and *A. foliolosus* is now understood to be driven primarily by herbivore avoidance rather than direct mutualism. Following *V. guaxarensis* discovery in 2020 (Marrero-Gómez *et al.*, 2020), a protective fence was installed around its range in Guajara. Since then, individuals have established independently without association with the Canary Island flatpod (Gesplan, 2019), further reinforcing previous studies on the importance of herbivore exclusion in shaping population dynamics (Seguí *et al.*, 2017).

Moreover, recent population growth may be mitigating genetic effects of herbivory. Between 2003 and 2019, violet populations in Teide and Guajara have grown from 10 040 individuals to 23 729 ± 4683 (Docoito Díaz, 2019). Specifically, the fenced T4 subpopulation (T4C), the only one protected from herbivores at the time of sampling, maintains both high density and the highest rates of genetic interaction observed in our results. Its large population size may buffer against the genetic erosion caused by herbivory in other areas; however, further research is needed to confirm this. Studies on the effects of herbivory on violet reproductive biology (Seguí *et al.*, 2017) provide a valuable foundation for simulation models to predict future impacts of herbivore predation on *V. cheiranthifolia* and *V. guaxarensis* in Teide and Guajara (Epps and Keyghobadi, 2015). This research, moreover, would be particularly relevant in the context of climate change, which is expected to increase rabbit distribution (Bello-Rodríguez *et al.*, 2021*b*).

### Gene flow patterns respond differently to environmental variables across species

We examined how landscape variables influence gene flow in both violet species and found contrasting patterns. In *V. cheiranthifolia*, gene flow declines with geographic distance, indicating isolation by distance (IBD). This is consistent with earlier findings of fine-scale genetic structure in the species (Rodríguez-Rodríguez *et al.*, 2019). The limited foraging range of insect pollinators, which tend to transfer pollen to nearby flowers, likely contributes to this pattern (Seguí *et al.*, 2017; Rodríguez-Rodríguez *et al.*, 2019). Similar IBD patterns have also been reported in other subalpine, insect-pollinated species in Teide National Park (Graham *et al.*, 2021). In addition, the violets’ restricted seed dispersal likely reinforces this genetic isolation by confining gene flow to short distances. In contrast, *V. guaxarensis* does not follow this IBD pattern. One likely explanation is the species’ dependency on non-natural seed establishment. In the presence of herbivores, *V. guaxarensis* populations were only established successfully within Canarian flatpod shrubs (*A. foliolosus*), which protect them from herbivory. This strict microhabitat requirement likely overrides distance-based dispersal constraints, decoupling geographic distance from gene flow.

Furthermore, in *V. guaxarensis*, the best-performing model was the dispersal vector model. Gene flow was higher across flatter slopes, suggesting that pollinators may prefer less steep terrain, possibly because gentle slopes facilitate easier foraging and movement (Makino, 2008). Temperature also played a role, as higher gene flow was associated with areas experiencing less diurnal temperature variation, likely due to enhanced pollinator activity under more stable thermal conditions. Likewise, gene flow increased with precipitation, which may be linked to improved seed establishment with humidity, as supported by previous studies (Seguí Colomar, 2017; Rodríguez-Rodríguez *et al.*, 2019). Although both violet species grow in poorly developed soils over lava flows, moisture retention does occur and depends on surface rock debris. This layer helps conserve water by reducing evaporation and protecting the substrate. In Guajara, where the litter is thinner and water retention lower, establishment may be more dependent on available water through precipitation (García Rodríguez, 2022). These findings suggest that environmental variables play a greater role than spatial distance in shaping potential gene flow in *V. guaxarensis*. However, these hypotheses require further empirical testing.

### Climate change does not change connectivity drastically

Our study identified only two climatic variables affecting connectivity significantly: BIO-02 (mean diurnal temperature range) and BIO-12 (annual precipitation), both relevant for *V. guaxarensis*. Projected changes in these variables are minimal: temperature shifts are less than 0.1 °C, and mean annual precipitation is expected to decrease by ∼5 mm (Supplementary Data Fig. S2), consistent with previous studies anticipating reduced precipitation (Martín Moreno, 2011; Bello-Rodríguez *et al.*, 2023). At the sites where *V. guaxarensis* occurs, these changes are very small, which likely explains why future climate scenarios have only minor predicted effects on genetic connectivity. For *V. cheiranthifolia*, no change is expected, as geographic distance, which strongly influences connectivity according to our analysis, is not projected to vary.

While projections suggest relatively stable connectivity patterns, this does not imply that these species are safe from climate-related threats. Habitat reduction from shifting climatic niches poses a direct threat and significant challenge to both species (Bello-Rodríguez *et al.*, 2023; Sarmiento Cabello *et al.*, submitted). Furthermore, climate change is expected to impact pollinator behaviour and abundance, critical components of gene flow not explicitly captured in our models. This is particularly concerning as it may disproportionately affect specialist interactions (Miller-Struttmann *et al.*, 2015; Lara-Romero *et al.*, 2019), upon which these violets rely. Combined with high selfing rates and limited seed dispersal, these unmodelled factors could lead to reduced reproductive success, dispersal and genetic diversity over time.

### Broader implication

This study highlights the power of gravity models as a valuable tool for estimating gene flow in endangered plant species, particularly those like the Canarian violets that exhibit low genetic structure and restricted spatial distributions. By enabling individual, interaction-based analyses, gravity models increase statistical power even in species with small sample size. Their exponential nature of connections also provides a more nuanced understanding of connectivity patterns, making them especially suited for identifying dispersal dynamics in rare or declining species. Thus, this modelling framework offers a scalable and replicable approach for landscape genetic studies in conservation biology.

Beyond methodological advances, our findings have direct conservation implications. By testing the impact of factors observed in the field that affect the species (e.g. herbivory pressure), their influence on gene flow and connectivity can be evaluated. Understanding these patterns enables the identification of source and sink populations, helping to prioritize areas for restoration and replantation. This is crucial for designing effective management strategies in ecosystems under pressure, especially when resources are limited. Furthermore, identifying conservation measures (e.g. population reinforcement) and geographic corridors that sustain genetic exchange is crucial for promoting long-term species persistence and ecological resilience, a central goal of contemporary conservation policy (Rico *et al.*, 2023). Our approach also aligns with national park priorities in territorial planning (Sarmiento Cabello *et al.*, 2026), providing a framework for evidence-based intervention in protected areas.

Looking forward, future research should integrate biotic interactions (such as consumer–resource interactions) and species traits alongside environmental predictors to provide a more comprehensive understanding of gene flow. Furthermore, temporal genetic analyses could help disentangle historically from contemporary connectivity patterns, offering insights into long-term responses to environmental change or conservation measures. Applying these findings to management actions, such as identifying replantation zones in emerging suitable habitats and designing ecological corridors, can directly support conservation goals under climate change. Ultimately, this work contributes to proactive, informed conservation planning that balances ecological needs with real-world constraints.

### Conclusions

This study provides the first comprehensive landscape genetics assessment of any plant species in the Canary Islands. Our study highlights the intricate interplay between environmental factors and genetic dynamics in *V. cheiranthifolia* and *V. guaxarensis*. Notably, *V. cheiranthifolia* exhibits higher genetic diversity at lower elevations. We observe that larger populations are associated with higher connectivity. In this context, we show that conservation measures increasing population density (e.g. herbivory exclusion) most likely positively impact connectivity. Moreover, these findings underscore the pivotal role of landscape features such as distance, slope, precipitation and temperature in shaping genetic connectivity within these species as well as consistent gene flow patterns for both species under future climate scenarios.

## Supplementary Material

mcaf317_Supplementary_Data

## Data Availability

The code used in this study is available on GitHub at the following repository: https://github.com/sonisarm/grav-models-violet.git. For further inquiries, please contact Sonia Sarmiento Cabello; sonia.sarmiento@ulpgc.es

## SUPPLEMENTARY DATA

Supplementary data are available at *Annals of Botany* online and consist of the following. Table S1: number of genetic samples analysed for *Viola cheiranthifolia* from Teide Mountain and *Viola guaxarensis* from Guajara, Tenerife, Canary Islands. Table S2: environmental variables considered in this study, with a spatial resolution of 20 m. Table S3: linear regression results showing the relationship between genetic diversity in six populations of *V. cheiranthifolia* and environmental variables. Table S4: collinearity between variables in 20-m buffer. The upper triangle shows the values for *V. cheiranthifolia* while the lower triangle shows values for *V. guaxarensis.*  Table S5: selected models and gravity model results for *V. cheiranthifolia* and *V. guaxarensis*. Table S6: number of pairwise connections across different gene flow value ranges in *V. cheiranthifolia* populations, and proportion of interactions (interactions per individual) of spatial sectors within Guajara population for *V. guaxarensis* (20-m buffer). Table S7: effect size of environmental variables from high-scoring models. Figure S1: map illustrating the functional connectivity of connections among nine subpopulations of *V. cheiranthifolia* for the 260-m buffer considering present conditions (left) and difference in gene flow between present conditions and scenario representative concentration pathway (RCP) 8.5 in year 2080 (right). Figure S2: histograms showing changes in climatic variables that significantly influenced gene flow in the top-performing model for *V. guaxarensis.*
