# Supplementary material for: Population density influences genetic connectivity in the Canary Islands endemics Viola cheiranthifolia and Viola guaxarensis
Source: Ann Bot. 2025 Dec 9;138(1):184–98. doi: 10.1093/aob/mcaf317 (PMC13409147; doi:10.1093/aob/mcaf317)
Supplement: mcaf317_Supplementary_Data [file mcaf317_supplementary_data.docx]

**Supplementary material**

**Population density drives genetic connectivity in the Canary Islands endemics *Viola cheiranthifolia* and *Viola guaxarensis***

Sonia Sarmiento Cabello[1](#_bookmark0),[*](#_bookmark3), Melanie Murphy[2](#_bookmark2), , Pedro A. Sosa[1](#_bookmark0), Alejandro G. Fernández de Castro[3](#_bookmark1), Jose Luis Martín Esquivel[4](#_bookmark2) and Priscila Rodríguez-Rodríguez[1](#_bookmark0)

*^1^Instituto Universitario de Estudios Ambientales y Recursos Naturales (IUNAT), Universidad de Las Palmas de Gran Canaria, Campus Universitario de Tafira, 35017 Las Palmas de Gran Canaria, Spain,*

*^2^Department of Ecosystem Science and Management/Program in Ecology and Evolution, University of Wyoming,*

*^3^Departamento de Biodiversidad y Conservación, Real Jardín Botánico – CSIC, calle Claudio Moyano, Madrid, Spain.*

*^4^Parque Nacional del Teide, C/. Dr. Sixto Perera González, 25, 38300. La Orotava, Tenerife, Spain,*

* Correspondence: sonia.sarmiento@ulpgc.es (S.S.C.)

E-mail [sonia.sarmiento@ulpgc.es](mailto:sonia.sarmiento@ulpgc.es)

Tel.: +34 928 454543; Fax: + 34 928 452922

**Table S1**: Number of genetic samples analyzed for *Viola cheiranthifolia* from Teide Mountain and *Viola guaxarensis* from Guajara, Tenerife, Canary Islands. The UTM coordinates indicate the population centroids, and the corresponding elevations were obtained from a 5-m resolution Digital Elevation Model (DEM) provided by the Instituto Geográfico Nacional (IGN), accessible via <https://martingonzalez.net/ign-dem-grabber/>.

| **Code area** | **Population code** | **Description** | **N** | **X** | **Y** | **Elevation (m)** |
| --- | --- | --- | --- | --- | --- | --- |
| ***Viola cheiranthifolia* (N=163)** | | | | | | |
| FOR | FOR | Rambleta Mirador Fortaleza | 5 | 339278.3 | 3128691 | 3,525 |
| MB | MBN | Montaña Blanca (North) | 16 | 341897.9 | 3128692 | 2,590 |
|  | MBS | Montaña Blanca (South) | 48 | 341478 | 3128257 | 2,721 |
| PV | PV | Pico Viejo | 19 | 337631.2 | 3127364 | 3,070 |
| RB | RB | Rambleta | 14 | 339088.1 | 3128610 | 3,592 |
| REF | REF | Refugio | 25 | 340196.5 | 3128648 | 3,264 |
| T4 | T4C | T4 (inside the fence) | 14 | 339249.7 | 3128034 | 3,488 |
|  | T4 | T4 | 12 | 339268.9 | 3128097 | 3,501 |
|  | T4E | T4 East | 10 | 339401.5 | 3128303 | 3,510 |
| ***Viola guaxarensis* (N=48)** | | | | | | |
| GUA | GUAS | Guajara (South) | 34 | 341804.3 | 3122106 | 2,679 |
|  | GUAN | Guajara (North) | 14 | 341761.8 | 3122302 | 2,637 |

**Table S2**: Environmental variables considered in this study, with a spatial resolution of 20 meters.

| **Description** | **Predictor variables** | **Origin** |
| --- | --- | --- |
| **Wordclim variables** | BIO-01, BIO-02, BIO-03, BIO-05, BIO-06, BIO-08, BIO-09, BIO-10, BIO-11, BIO-12, BIO-13, BIO-14, BIO-15, BIO-16, BIO-17, BIO-18 | Minimum, average and maximum temperature and total precipitation with the procedure established by González Fernández de Castro (2016) from 275 climatic stations in the Tenerife agro-climatic network |
| **Topographic variables** | Slope, Topographic Position Index (TPI), northness | Instituto Geográfico Nacional (IGN) |
| **Snow cover** | | Acquired by Landsat images |
| **Radiation** | | Agencia Estatal de Meteorología (AEMET) |
| **Potential Evapotranspiration (PET)** | | *r2dRue* package in R using temperature, radiation and soil type |

**Table S3**: Linear regression results showing the relationship between genetic diversity in six populations of *V. cheiranthifolia* and environmental variables. The values are ordered alphabetically and effect size (t-value). Abbreviations. PET: potential evapotranspiration. TPI: Topographic Position Index.

| **Variable** | **Estimate** | **Standard Error** | **T-value** | **P-value** |
| --- | --- | --- | --- | --- |
| PET | 0.0046 | 0.0020 | 2.3312 | 0.0801 |
| radiation | 0.0000 | 0.0000 | 2.1071 | 0.1028 |
| BIO-06 | 0.1917 | 0.0946 | 2.0278 | 0.1125 |
| BIO-11 | 0.1883 | 0.0951 | 1.9796 | 0.1189 |
| BIO-01 | 0.2111 | 0.1073 | 1.9684 | 0.1204 |
| BIO-10 | 0.1980 | 0.1009 | 1.9620 | 0.1213 |
| BIO-05 | 0.1473 | 0.0794 | 1.8545 | 0.1373 |
| BIO-12 | 0.0074 | 0.0042 | 1.7441 | 0.1561 |
| BIO-16 | 0.0161 | 0.0098 | 1.6493 | 0.1744 |
| BIO-09 | 0.1277 | 0.0789 | 1.6187 | 0.1808 |
| BIO-08 | 0.1262 | 0.0796 | 1.5860 | 0.1879 |
| BIO-18 | 0.0519 | 0.0376 | 1.3806 | 0.2395 |
| BIO-17 | 1.1258 | 0.8377 | 1.3439 | 0.2501 |
| BIO-02 | 1.9497 | 2.9524 | 0.6604 | 0.5451 |
| northness | -0.2732 | 0.2856 | -0.9565 | 0.3930 |
| BIO-03 | -3.3566 | 3.1096 | -1.0794 | 0.3411 |
| slope | -2.2538 | 1.8069 | -1.2473 | 0.2803 |
| snow | 0.0000 | 0.0000 | -1.4397 | 0.2234 |
| BIO-13 | -0.1132 | 0.0782 | -1.4472 | 0.2214 |
| TPI | -0.5523 | 0.3701 | -1.4923 | 0.2099 |
| BIO-15 | -0.0142 | 0.0077 | -1.8452 | 0.1388 |

**Table S4:** Collinearity between variables in 20-m buffer. The upper triangle shows the values for *V. cheiranthifolia* while the lower triangle shows values for *V. guaxarensis.* Variables are labeled according to their role in the gravity model: as weights (*w*), at-site variables (*v*), or resistance values (*c*). Values above 0.7 are highlighted.

| **Variables** | length (*w*) | BIO-01 (*v*) | Slope (*v*) | snow cover (*v*) | BIO-02 (*c*) | BIO-12 (*c*) | Slope (*c*) | Radiation (*c*) |  |
| --- | --- | --- | --- | --- | --- | --- | --- | --- | --- |
| *V. cheiranthifolia* (upper) and *V. guaxarensis* (lower) | | | | | | | | | |
| length (*w*) | 1 | 0.482 | -0.215 | -0.242 | 0.642 | 0.528 | 0.680 | 0.288 |  |
| BIO-01 (*v*) | 0.220 | 1 | -0.671 | -0.443 | 0.461 | 0.617 | 0.268 | 0.046 |  |
| Slope (*v*) | 0.251 | 0.685 | 1 | 0.629 | -0.194 | -0.450 | -0.135 | -0.172 |  |
| snow cover (*v*) | 0.272 | 0.640 | **0.872** | 1 | -0.331 | -0.197 | -0.145 | -0.345 |  |
| BIO-02 (*c*) | -0.571 | -0. 071 | -0.371 | -0.455 | 1 | 0.539 | 0.389 | 0.349 |  |
| BIO-12 (*c*) | -0.474 | 0. 376 | 0.209 | 0.185 | 0.486 | 1 | 0.293 | -0.134 |  |
| Slope (*c*) | 0.107 | 0. 511 | 0.612 | 0.635 | -0.384 | 0.572 | 1 | 0.317 |  |
| Radiation (*c*) | -0.005 | -0. 480 | -0.575 | -0.617 | 0.331 | -0.636 | **-0.983** | 1 |  |

| **Table S5**: Selected models and gravity model results for *V. cheiranthifolia* and *V. guaxarensis*. Abbreviations: np= number of parameters; nl=Snow cover. Models with ΔAIC < 4 are highlighted. | | | | | | | | | |
| --- | --- | --- | --- | --- | --- | --- | --- | --- | --- |
| **Hypothesis** | **Model (*v*)** | **Model (*c*)** | **AIC** | **BIC** | **lh (log)** | **RMSE** | **nparms** | **ΔAIC** | **ΔBIC** |
| *Viola cheiranthifolia* (20-m buffer) | | | | | | | | | |
| **Null** | - | - | -349.951 | -340.845 | 178.9757 | 1.1302 | 2 | 0 | 0 |
| **Habitat suitability** | BIO-01 + nl + Slope | - | -346.12 | -330.183 | 180.0599 | 1.1301 | 5 | 3.831501 | 10.6615 |
| **Establishment** | - | BIO-12 + Slope | -348.306 | -334.646 | 180.153 | 1.1302 | 4 | 1.645281 | 6.198613 |
| **Topography** | Slope | Slope | -349.162 | -335.502 | 180.5812 | 1.1302 | 4 | 0.789069 | 5.342402 |
| Temperature | BIO-01 | BIO-02 + Radiation | -344.113 | -328.177 | 179.0567 | 1.1302 | 5 | 5.838049 | 12.66805 |
| **Water** | nl | BIO-12 | -346.052 | -332.392 | 179.0262 | 1.1302 | 4 | 3.898996 | 8.452329 |
| Edge | - | BIO-02 + BIO-12 + Slope + Radiation | -344.343 | -326.13 | 180.1716 | 1.1302 | 6 | 5.608151 | 14.71482 |
| Global | BIO-01 + nl + slope | BIO-02 + BIO-12 + Slope + Radiation | -340.975 | -315.932 | 181.4877 | 1.1301 | 9 | 8.976018 | 24.91268 |
| *Viola cheiranthifolia* (260-m buffer) | | | | | | | | | |
| **Null** | - | - | -349.951 | -340.845 | 178.9757 | 1.1302 | 2 | 0 | 0 |
| **Habitat suitability** | BIO-01 + nl + Slope | - | -346.12 | -330.183 | 180.0599 | 1.1301 | 5 | 3.831501 | 10.6615 |
| **Establishment** | - | BIO-12 + Slope | -348.671 | -335.011 | 180.3353 | 1.1302 | 4 | 1.280792 | 5.834124 |
| **Topography** | Slope | Slope | -349.351 | -335.691 | 180.6755 | 1.1302 | 4 | 0.600367 | 5.153699 |
| Temperature | BIO-01 | BIO-02 + Radiation | -344.3 | -328.363 | 179.1499 | 1.1302 | 5 | 5.651637 | 12.48164 |
| **Water** | nl | BIO-12 | -346.047 | -332.387 | 179.0233 | 1.1302 | 4 | 3.904728 | 8.45806 |
| Edge | - | BIO-02 + BIO-12 + Slope + Radiation | -344.686 | -326.473 | 180.3432 | 1.1302 | 6 | 5.264898 | 14.37156 |
| Global | BIO-01 + nl + slope | BIO-02 + BIO-12 + Slope + Radiation | -341.325 | -316.282 | 181.6627 | 1.1301 | 9 | 8.62594 | 24.5626 |
| *Viola guaxarensis* | | | | | | | | | |
| Null | - | - | -2358.43 | -2337.88 | 1183.217 | 1.1321 | 2 | 136.3557 | 120.9391 |
| Habitat suitability | BIO-01 + Slope | - | -2363.61 | -2332.77 | 1187.803 | 1.1318 | 4 | 131.1832 | 126.0443 |
| Establishment | - | BIO-12 + Slope | -2427.47 | -2396.64 | 1219.735 | 1.1316 | 4 | 67.32031 | 62.18144 |
| Topography | Slope | Slope | -2426.7 | -2395.87 | 1219.352 | 1.1316 | 4 | 68.08516 | 62.9463 |
| Temperature | BIO-01 | BIO-02 | -2365.27 | -2334.43 | 1188.633 | 1.1318 | 4 | 129.5232 | 124.3843 |
| **Edge** | - | BIO-02 + BIO-12 + Slope | -2494.79 | -2458.82 | 1254.395 | 1.1312 | 5 | 0 | 0 |
| **Global** | BIO-01 + slope | BIO-02 + BIO-12 + Slope | -2491.51 | -2445.26 | 1254.755 | 1.1312 | 7 | 3.278993 | 13.55673 |

**Table S6:** Number of pairwise connections across different gene flow value ranges in *Viola cheiranthifolia* populations, and proportion of interactions (interactions per individual) of spatial sectors within Guajara population for *Viola guaxarensis* (20-m buffer)

| ***Viola cheiranthifolia*** | | | |
| --- | --- | --- | --- |
| **Population** | **0.455 - 0.47** | **0.471 - 0.48** | **0.481 - 0.49** |
| FOR | 8 | 0 | 0 |
| MBN | 8 | 0 | 0 |
| MBS | 8 | 0 | 0 |
| PV | 8 | 0 | 0 |
| RB | 0 | 7 | 0 |
| REF | 1 | 7 | 0 |
| T4 | 6 | 1 | 0 |
| T4C | 0 | 6 | 2 |
| T4E | 4 | 4 | 0 |
| ***Viola guaxarensis*** | | | |
| **Population** | **0.38-0.45** | **0.451-0.48** | **0.481-0.55** |
| GUAS | 8.2 | 54.88 | 2.77 |
| GUAN | 13 | 15.54 | 1.22 |

**Table S7**: Effect size of environmental variables from high-scoring models. Abbreviations: df = degrees of freedom; CI = confidence interval.

| **Variable** | **T value** | **Cohen’s D** | **low CI** | **up CI** |
| --- | --- | --- | --- | --- |
| ***Viola cheiranthifolia*** | | | | |
| BIO-01 | –0.761 | –0.681 | –2.370 | 1.008 |
| Snow cover | –0.060 | –0.034 | –1.485 | 1.417 |
| Slope (v) | –0.840 | –0.660 | –2.030 | 0.709 |
| Geographic distance | -2.519 | -0.644 | -1.014 | -0.274 |
| BIO-12 | 0.177 | 0.045 | –0.318 | 0.409 |
| Slope (c) | 1.391 | 0.357 | –0.008 | 0.723 |
| ***Viola guaxarensis*** | | | | |
| BIO-01 | –0.570 | –0.199 | –0.699 | 0.302 |
| Slope (v) | 0.292 | 0.102 | –0.398 | 0.601 |
| Geographic distance | –0.0738 | –0.042 | –0.121 | 0.038 |
| BIO-02 | –8.306 | –0.475 | –0.556 | –0.395 |
| BIO-12 | 7.674 | 0.439 | 0.359 | 0.519 |
| Slope (c) | -10.752 | -0.616 | -0.697 | -0.534 |


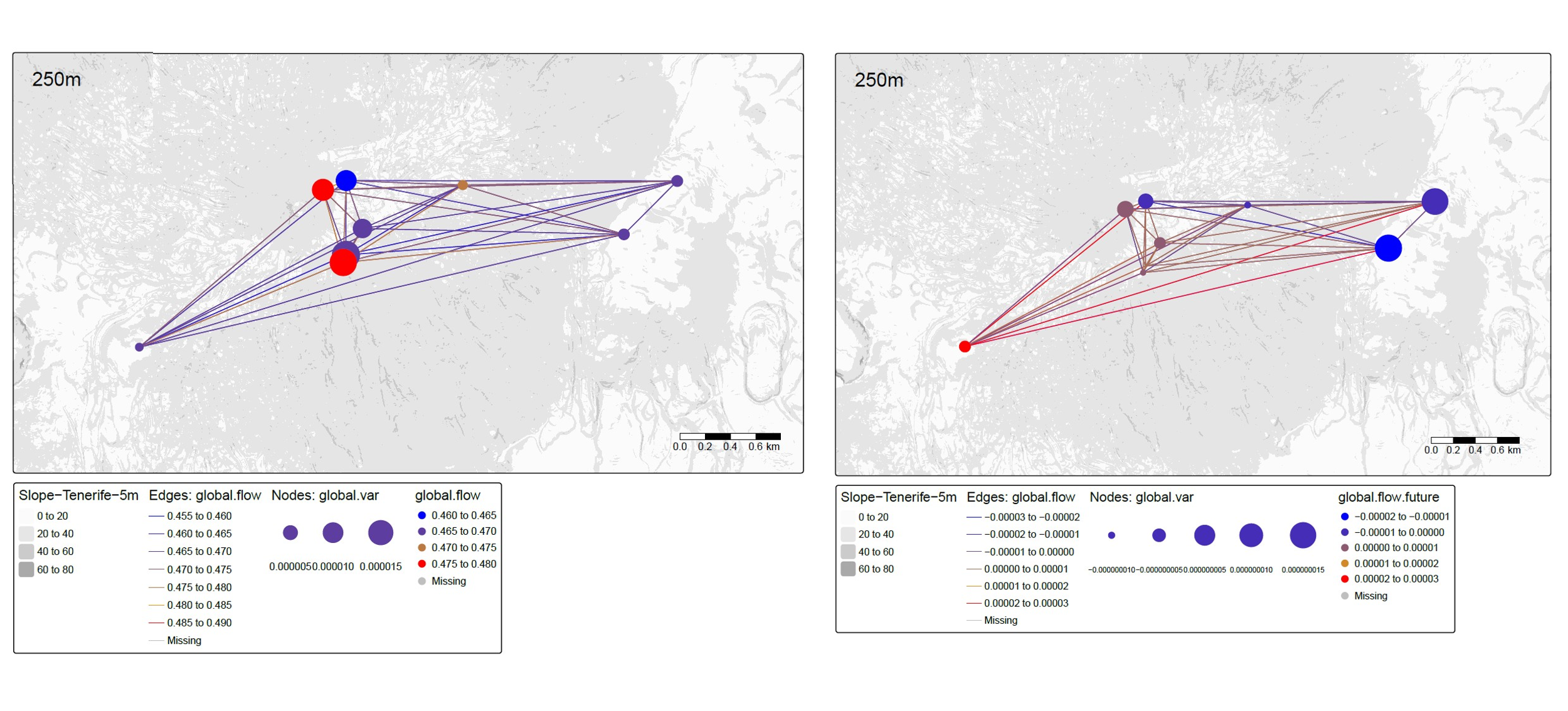


**Figure S1:** Map illustrating the functional connectivity of connections among 9 subpopulations of V. cheiranthifolia for 260-m buffer considering present conditions (left) and difference in gene flow between present conditions and scenario Representative Concentration Pathway (RCP) 8.5 in year 2080 (right). Gene flow values (0-1) are indicated between (edges) and within sites (nodes), as well as global variance at node.


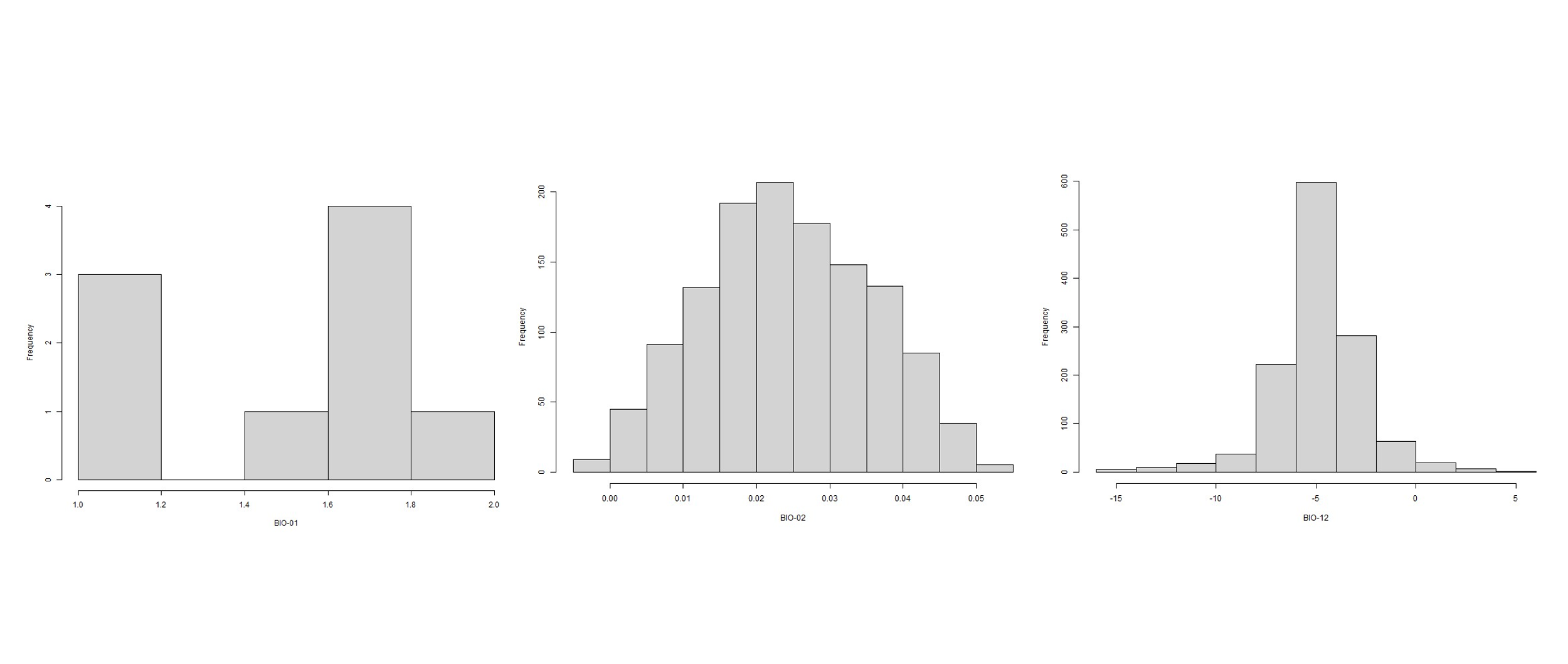


**Figure S2.** Histograms showing changes in climatic variables that significantly influenced gene flow in the top-performing model for *V. guaxarensis.* Changes in mean diurnal temperature variation (BIO-02) and annual mean precipitation (BIO-12) are based on between-site values across over 1,200 pairwise interactions. All comparisons reflect projected differences between present-day conditions and the Representative Concentration Pathway (RCP) 8.5 climate scenario for the year 2080.
